# Use of predictive tools in the management of COVID-19 patients: a key role of clinical laboratories

**DOI:** 10.1515/almed-2020-0059

**Published:** 2020-10-29

**Authors:** Carla Martín Grau, Clara Benavent Bofill, Ester Picó-Plana, Gemma Recio Comí, Margarida Terrón-Puig, Natalia Bastón Paz, MaTeresa Sans Mateu, Cristina Gutiérrez Fornés

**Affiliations:** Clinical Chemistry Laboratory, Catalan Institute of Health (ICS)- Camp de Tarragona-Terres de l’Ebre, Joan XXIII University Hospital in Tarragona, Tarragona, Spain; and Institut d´Investigació Sanitària Pere Virgili, Joan XXIII University Hospital in Tarragona, Tarragona, Spain; and Clinical Chemistry Laboratory, Catalan Institute of Health (ICS)- Camp de Tarragona-Terres de l’Ebre, Verge de la Cinta Hospital in Tortosa, Tarragona, Spain

**Keywords:** COVID-19, laboratory management, lactate dehydrogenase (LDH)**-**to**-**leukocyte ratio, molecular diagnostic test, predictive tool, technical resources

## Abstract

**Objectives:**

Coronavirus disease 2019 (COVID-19) is widely spreading and represents a critical threat to global health. In the fight against this pandemic, provincial hospitals urgently need rapid diagnostic of COVID-19 infected patients to avoid collapsing of emergency units. However, the high demand of patients with severe acute respiratory symptoms limits the fast delivery of results by the gold standard method reverse transcription-polymerase chain reaction real time (rRT-PCR) for the identification of COVID-19 positive pneumonia. The principal aim is to find other useful laboratory indicators to assist rRT-PCR tests and to help controlling of this outbreak.

**Methods:**

Blood, coagulation and inflammatory parameters were collected from a total of 309 patients classified as negative (128) and positive (181) rRT-PCR test groups. Patients were classified as positive by molecular diagnostic test.

**Results:**

Leukocyte count (WBC), neutrophils count, lymphocytes count and lactate dehydrogenase (LDH) were statistically different between both groups of patients. The use of LDH/WBC ratio increases the diagnostic performance with the best area under the curve (0.783), sensibility (82%) and the best percentage (80.5%) of correctly identified COVID-19 positive patients.

**Conclusions:**

The combination of predictive LDH/WBC ratio with clinical illness features could help in medical management of patients and improve the technical resources of hospitals, especially in a critical scenario with a large shortage of medical equipment and lack of reagents for performing rRT-PCR.

## Introduction

Coronavirus disease 2019 (COVID-19) was identified by the Chinese Centre for Disease Control and Prevention (CDC) from the throat swab sample of patients with pneumonia of unknown cause [1], and was subsequently named 2019-nCoV by WHO [2]. Since December 2019, COVID-19 is widely spreading and represents a critical threat to global health due to its high incidence and infectivity. Up to the date of May 21st, 2020, around 5,121,639 people have been infected and 333,323 have died [3]. In the fight against this worldwide pandemic, rapid identification of clinical and laboratory predictors of diagnostic and progression toward severe and fatal forms is urgently needed. These predictors are necessary in some Hospital Departments, such as an Emergency Department, for risk stratification, optimization of patient’s allocation in specific areas and correct use of human and technical resources in the course of the pandemic. Hence, laboratory parameters have demonstrated to be capable of discriminating between severe and nonsevere cases [4], [5], [6], as well as determine the risk of mortality [7]. Hospitals must mainly focus on the correct identification of COVID-19 disease in patients and implement ways to stop or slow down the disease progression. Nevertheless, the early intervention depends on the results of clinical laboratory and the gold standard method for the etiological diagnosis of COVID-19 is reverse transcription-polymerase chain reaction real time (rRT-PCR) test with long turnaround times (on average over 3–5 h to generate results). Furthermore, hospitals are facing several limitations such as laboratories certification, expensive equipment, trained personnel [8] and appropriate measures to keep laboratory staff safe while producing reliable test results [9]. At present, the high volume of patients with severe acute respiratory symptoms who arrive at the Emergency Department is highlighting the limitations of rRT-PCR test and emphasizes the use of alternative tests to quickly identify infected patients at the time of admission for the subsequent management of those cases.

In Tarragona, Catalonia province, Spain, up to date 17,277 rRT-PCR have been performed to supposedly infected patients in the Clinical Chemistry Laboratory. The number of determinations has increased more than 1.200% compared to a normal year without COVID-19. Severe difficulties were presented due to the shortage of reagents that led to the collapse of certain Medical Departments on account of delayed results. Provincial hospitals do not have enough areas to locate undiagnosed patients; so it is important to have the results as quickly as possible in order to organize admissions. Given this scenario, it is urgent to find accessible diagnostic measures.

The aim of this study is to develop and validate the application of predictive tools that discriminate between positive and negative COVID-19 patients in order to prevent virus transmission in hospital areas and guarantee correct measures in patients monitoring and treatment. The utility of combining powerful laboratory parameters could help in medical management of patients and improve the human and technical resources of the hospital, especially in those hospital centers that have suffered a large shortage of medical equipment and have difficulty receiving reagents for performing rRT-PCR.

## Materials and methods

### Patients and sample collection

A total of 309 anonymized cases over 18 years-old (129 women and 180 men) were included in this study and classified into two groups based on the results of rRT-PCR: negative (128) and positive (181) rRT-PCR test groups. Patients were classified as positive when *E* gene (screening gene) was <35 cycle threshold (Ct) or *E* gene was >35 Ct with *N* gene (confirmatory gene) <40 Ct. In case of lack, the *RdRp* gene was used as confirmatory gene. All patients were admitted to Joan XXIII University Hospital (Tarragona, Spain) Emergency Department between the 13th of March and 21st of May of 2020 as potential COVID-19 cases who presented fever and initial respiratory signs [10].

Only anonymized data obtained from routine diagnostic analysis was used for statistical purposes in this study. No extra experiments were performed; for this reason, no specific informed consent was required for this study. Therefore, it is exempted from approval by the Institutional Review Board.

Upper respiratory specimens: nasopharyngeal/oropharyngeal swab were collected in tubes containing standard viral transport medium (DeltaSwab ViCUM^®^, Deltalab, Barcelona, Spain) and/or lower respiratory specimens: sputum (if produced) and/or endotracheal aspirate or bronchoalveolar lavage in patients with more severe respiratory disease were collected in sterile tubes (EUROTUBO^®^, Deltalab, Barcelona, Spain). All samples were collected according to WHO guidelines [11]. Blood samples were collected in EDTA anticoagulant tubes and without anticoagulant tubes (Vacutainer, Becton Dickinson, Rutherford, NJ, USA).

Blood sample evaluation was performed the same day of the rRT-PCR test and included a full blood count in the automated analyzer Sysmex XN-9000^®^ (Sysmex Corporation, Kobe, Japan), measurement of C-reactive protein, lactate dehydrogenase (LDH), creatinine and ferritin assessed using ADVIA^®^ Chemistry XPT analyzer (Siemens Healthcare Diagnostics Inc., Tarrytown, NY, USA). Also, measurement of high-sensitivity troponin I (TNIH) and N-terminal pro-B-type natriuretic peptide (NT-proBNP) was in ADVIA Centaur^®^ XPT Immunoassay analyzer (Siemens Healthcare Diagnostics Inc., Tarrytown, NY, USA). D-dimers and fibrinogen were performed by ACL TOP 500 CTS^®^ (Werfen, Barcelona, Spain). The rRT-PCR was performed on a CFX96 Touch System thermocycler (Bio-Rad Laboratories Inc., Hercules, California, USA) using a commercial kit that targeted *E*, *N* and *RdRp* genes (LightMix^®^ Modular SARS and Wuhan CoV, TIB MOLBIOL, Berlin, Germany). RNA purification was obtained using the RNeasy Mini Kit in the automated analyzer QIAcube Connect (QIAGEN, Hilden, Germany).

### Statistical analysis

Quantitative data were described as mean ± SD for normally distributed data. Median and interquartile range (IQR) were used for the data that were not normally distributed. For comparison between groups an unpaired Student’s t-test was used. A Mann-Whitney U test was used for the data that were not normally distributed. For categorical variables, Fisher’s exact test was used. To analyze the differences in rRT-PCR results according to patients’ demographics factors, a one-way analysis of variance (ANOVA) was used for variables with more than two categories (age range).

The diagnostic accuracy of each laboratory parameter was assessed by using Youden’s index (YI), sensitivity, specificity, positive and negative predictive values that were calculated as follows: YI = sensitivity + specificity – 100; sensitivity = true positive/(true positive + false negative); specificity = true negative/(true negative + false positive); positive predictive value = true positive/(true positive + false positive); negative predictive value = true negative/(true negative + false negative). The Youden’s index is a function of sensitivity and specificity that depends on the underlying distributions of the diseased (i.e., positive rRT-PCR test group) and non-diseased (i.e., negative rRT-PCR test group) populations and measures the effectiveness of a diagnostic marker (i.e.,) as well as enables the selection of an optimal threshold value (cut-off point) for the marker [12]. Furthermore, receiver operative characteristic (ROC) curves were constructed to calculate the area under the curve (AUC) for each index.

The collected data were processed using the statistical software package SPSS version 25.0 for Windows (SPSS, Chicago, IL, USA). A p-value <0.05 was considered statistically significant.

## Results

This study includes the analytical parameters of 309 anonymized patients with 128 negative rRT-PCR test result (median age of 69 years, range 20–69) and 181 positive rRT-PCR test result (median age of 70 years, range 19–93). The demographic data of the two groups are shown in Table 1. There were no significant differences between positive and negative rRT-PCR test group regarding to age (p=0.751). Nevertheless, comparing by age range, significant differences are observed with respect to the Ct of the *E* (p<0.0001) and *N* (p=0.002) genes detected in positive rRT-PCR test group. Patient with >70 years demonstrated less Ct than other age ranges. Additionally, according to the sex of the patient (p=0.027) significant values were found in positive patients (115 males, 63.5%) when compared to negative rRT-PCR test group (65 males, 50.8%).

**Table 1:** Demographics of patients admitted to the Emergency Department of University Joan XXIII Hospital (March 13th to May 21st 2020).

	Positive rRT-PCR test group^a^ (n=181)				Negative rRT-PCR test group^b^ (n=128)	Significance p-value (between^a,b^)
		Gene (Ct)^d^				
		*E* (n=181) Mean ± SD	*R* (n=81) Mean ± SD	*N* (n=114) Mean ± SD		
Age, years	70 (19–93)				69 (20–96)	0.751
Age range^c^						
≤39	13 (7.2%)	30.20 ± 7.68	29.02 ± 9.33	31.63 ± 6.60	13 (10.2%)	
40–49	17 (9.4%)	31.03 ± 5.05	33.83 ± 1.75	34.32 ± 3.06	17 (13.3%)	
50–59	35 (19.3%)	30.60 ± 4.93	30.02 ± 5.18	31.70 ± 6.78	16 (12.5%)	
60–69	22 (12.2%)	26.97 ± 6.52	29.40 ± 5.21	30.36 ± 5.41	21 (16.4%)	
≥70	94 (51.9%)	25.02 ± 6.28	27.31 ± 6.15	27.53 ± 6.35	61 (47.7%)	
**p-Value (between** ^**c,d**^ **)**		<0.0001^e^	0.061	0.002^e^		
Sex						0.027^e^
Female	66 (36.5%)				63 (49.3%)	
Male	115 (63.5%)				65 (50.8%)	

SD, standard deviation; Ct, cycle threshold. For age, median and range have been presented due to the non-normality of data. Correspondingly, Mann-Whitney U test was used to assess if differences between positive^a^ rRT-PCR and negative^b^ rRT-PCR test group were statistically significant. One-way analysis of variance (ANOVA) was used to compare age range^c ^and gene detection^d^ by rRT-PCR. For sex, Fisher’s exact test was used to check if there is any association between positive^a^ rRT-PCR and negative^b^ rRT-PCR test group were statistically significant. ^e^Indicates p-values <0.05.

At the time of admission, a complete blood analysis and rRT-PCR test were performed to all patients presenting initial signs of COVID-19 infection. Table 2 shows all laboratory parameters tested. Precisely, a significant decrease in leukocyte count (WBC) (p<0.0001), neutrophils count (p<0.0001), lymphocytes count (p<0.0001) and platelets (p=0.045) were found in positive rRT-PCR test group when were compared to negative rRT-PCR test group. Even though, a significant increase in LDH (p<0.0001) was observed in positive rRT-PCR test group in contrast to negative rRT-PCR test group. However, no differences were detected in red blood parameters, coagulation function (D-dimers and fibrinogen), TNIH and NT-proBNP.

**Table 2:** Comparison of laboratory parameters between positive and negative rRT-PCR test groups.

Parameters	Positive rRT-PCR test group^a^ (n=181)		Negative rRT-PCR test group^b^ (n=128)		Significance p-value (between^a,b^)
		Mean ± SD		Mean ± SD	
Blood routine					
Red blood cells (RBC), ×10^6^/µL	n=176	4.16 ± 0.84	n=128	4.11 ± 0.76	0.846
Hemoglobin (Hb), g/dL	n=176	12.11 ± 2.13	n=128	12.03 ± 2.22	0.544
Hematocrit (Htc), %	n=176	37.29 ± 6.45	n=128	37.30 ± 6.39	0.809
Mean cell volume (MCV), fL	n=176	90.44 ± 7.43	n=128	91.22 ± 7.78	0.780
Mean corpuscular hemoglobin (MCH), pg	n=176	29.31 ± 2.65	n=128	29.38 ± 2.88	0.648
Mean corpuscular hemoglobin concentration (MCHC), g/dL	n=176	32.38 ± 1.42	n=128	32.19 ± 1.40	0.481
Red distribution width (RDW), %	n=176	14.12 ± 2.28	n=128	14.34 ± 1.97	0.474
		**Median (IQR)**		**Median (IQR)**	
Leukocytes (WBC), ×10^9^/µL	n = 176	6.50 (24.99)	n = 128	9.27 (23.50)	<0.0001^c^
Neutrophils, ×10^9^/µL	n=176	4.89 (21.44)	n=128	6.76 (23.32)	<0.0001^c^
Lymphocytes, ×10^9^/µL	n=176	1.01 (4.44)	n=128	1.38 (3.05)	<0.0001^c^
Platelets, ×10^9^/µL	n=176	201.5 (547)	n=128	223.5 (739)	0.045^c^
Coagulation function					
D-dimers, ng/dL	n=121	715.0 (67976)	n=26	520.50 (14012)	0.391
Fibrinogen, mg/dL	n=154	672.0 (1117)	n=107	631.0 (649)	0.074
Blood biochemistry					
C-reactive protein, mg/dL	n=164	6.45 (35)	n=125	6.30 (50.60)	0.519
Creatinine, mg/dL	n=174	0.83 (13.19)	n=126	0.88 (5.01)	0.810
Lactate dehydrogenase (LDH), U/L	n=139	248 (763)	n=58	209.50 (370)	<0.0001^c^
Ferritin, ng/mL	n=23	672.0 (4567)	n=7	249.0 (403)	0.077
High-sensitivity troponin I (TNIH), ng/L	n=103	10.0 (5218)	n=33	13.0 (23803)	0.092
N-terminal pro-B-type natriuretic peptide (NT-proBNP), pg/mL	n=14	642 (9224)	n=21	2683.0 (27312)	0.069

SD, standard deviation; IQR, interquartile range.

Student’s t-test was used for RBC, Hb, Htc, MCV, MCH, MCHC and RDW parameters. Mann-Whitney U test was used for WBC, neutrophils, lymphocytes, platelets, D-dimers, fibrinogen and all blood biochemistry analysis. Both statistic tests were used to assess if differences between positive^a^ rRT-PCR and negative^b^ rRT-PCR test group were statistically significant. ^c^Indicates p-values <0.05.

**Figure 1:**
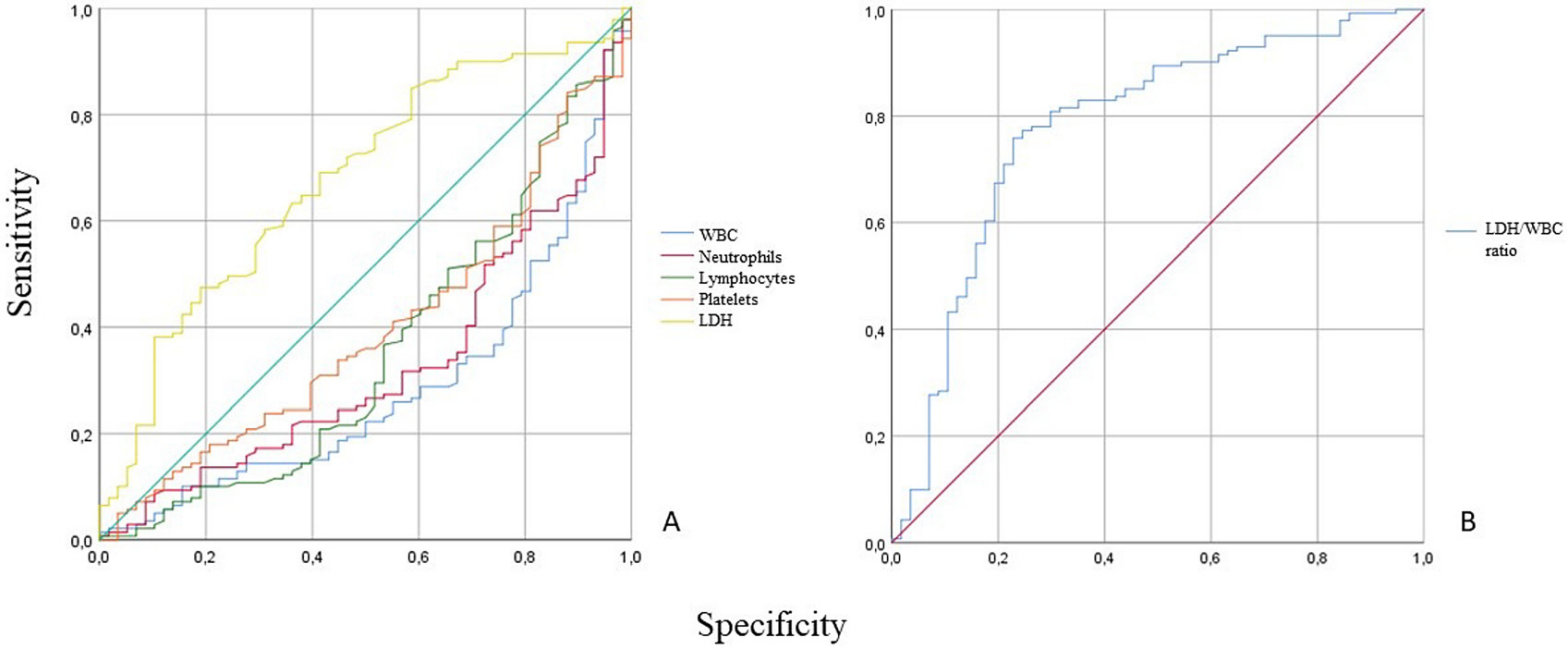
ROC curves of laboratory parameters (A) and LDH/WBC ratio (B) for differentiating positive from negative rRT-PCR test group.

Hence, considering the significant values obtained that differentiated positive and negative rRT-PCR test group, AUC, cut-off values, sensitivity, specificity and Youden’s index of efficient laboratory parameters were calculated in Table 3. Precisely in Figure 1A, WBC and LDH demonstrated the highest AUC (0.713 and 0.679, respectively). Further, WBC had the highest sensitivity among all parameters examined, 76%. The highest specificity, 81%, was demonstrated by LDH. Based on these results, we tested a new LDH/WBC ratio shown in Table 3. Cut-off values higher than 25.64 indicate positive rRT-PCR test group. The LDH/WBC ratio was a better effective tool with the highest AUC (0.783, Figure 1B) and sensitivity (82%) when was compared to other significant parameters. Also, the highest percentage of correctly identified patients (80.5%) was provided by the new ratio.

**Table 3:** The diagnostic accuracy of significative parameters used in this study and their cut-off to discriminate positive from negative rRT-PCR test group.

Parameters	AUC (95% CI)	Cut-off positive group	S, %	E, %	PPV, %	NPV, %	CP, %	Youden’s index, %
WBC	0.713 (0.635–0.791)	<7.24	76	63	71	81	76	39
Neutrophils	0.674 (0.591–0.758)	<5.25	69	65	71	77	74	34
Lymphocytes	0.643 (0.556–0.730)	<1.48	50	77	81	65	73	27
Platelets	0.602 (0.518–0.686)	<208.0	69	53	68	71	69.5	22
LDH	0.679 (0.598–0.760)	>260.50	48	81	66	83	74.5	29
LDH/WBC	0.783 (0.707–0.856)	>25.64	82	69	85	76	80.5	51

AUC, area under the curve; CI, confidence intervals; S, sensitivity; E, specificity; PPV, positive predictive value (%); NPV, negative predictive value (%); CP, correctly identified patients (%).

## Discussion

Human coronavirus is one of the main pathogens of respiratory infections. Patients with COVID-19 usually have a rapid progression of pneumonitis and even develop acute respiratory distress syndrome and death [13]. Among the 309 patients presented in the Emergency Department, all were enrolled in this study because of their suggestive symptoms of COVID-19 infection. rRT-PCR tests were performed in all cases to classify them into the positive and negative groups. A greater number of men (63.5%) than women was observed in the 181 positive rRT-PCR test group. Li et al. [14] and Guan et al. [15] have also found that males are more infected than females. Nanshan et al. [16] suggested that the reduced susceptibility of females to viral infection could be attributed to the protection from X chromosome and sex hormones, which play an important role in innate and adaptive immunity. Truly, COVID-19 has highlighted the vulnerability of aging populations to emerging diseases [17]. Critically, 51.9% of positive rRT-PCR test group was older than 70 years and presented the lowest Ct values for *E*, *R* and *N* genes than the other age ranges. Seemingly, high viral load (inversely related to Ct value) might be used for assessing disease severity and prognosis [18].

Notwithstanding the foregoing, the aim of this study was to develop and validate the application of predictive tools that discriminate between positive and negative COVID-19 patients in order to have the results as quickly as possible as a way to organize admissions and prevent virus transmission in hospital’s areas when rRT-PCR results could be delayed. In terms of laboratory tests, the values of WBC, neutrophils and lymphocytes counts are significatively reduced in positive rRT-PCR test group. In previous reports, low WBC and lymphocytes counts were found in positive patients [19], [20], [21], which is in line with our study but differed in neutrophil counts [16], [19]. In addition, lymphocyte count may serve as a clinical predictor of positive infection due to their role in the elimination of virally infected cells. COVID-19 viral particles damage the cytoplasmic component of the lymphocyte and lead to destruction [22]. Therefore, the capacity of an affected patient to replenish these cells is compromised [23]. Furthermore, it is assumed that after a viral infection, lymphocytopenia in peripheral blood is on account of lymphocyte sequestration firstly [24]. Xiong et al. [25] explain that neutrophil chemoattractant CXCL2 and CXCL8 facilitate the migration of these immune cells to the site of infection. It seems that the neutropenia observed in positive rRT-PCR tests group is owing to the cell infiltration in lung tissues of COVID-19 patients. Regarding to the coagulation function, platelets count was significantly different between the two groups. Thrombocytopenia at admission in positive COVID-19 patient was also observed by Liu et al. [13] as a prognosis predictor. The consumption of platelets is associated with the rise of D-dimers and fibrinogen [13], [26]. In this study, positive rRT-PCR test group had increased values of D-dimers and fibrinogen when compared to negative rRT-PCR test group ,but it was not statistically significant. Based on the findings of inflammatory and organ injury biomarkers, LDH had significantly increased in positive rRT-PCR test group. The mentioned value has demonstrated being an efficient tool in discriminating between patients infected or uninfected patients with COVID-19 [8] and as a predictive tool of severity [27]. No significative differences were found in PCR or ferritin, contrary to others studies previously published [8], [19].

In order to tentatively develop a predictive method based on routine blood test analysis, we highlight that WBC, neutrophils, lymphocytes count and LDH have a good accuracy in predicting positive rRT-PCR cases. Precisely, WBC and LDH had the best AUC (0.713 and 0.679, respectively) that promoted to use of the LDH/WBC ratio with the consequent improvement of the AUC (0.783) and sensitivity (82%). This ratio obtained a high percentage of correctly identification of patients (80.5%) before performing the rRT-PCR test. Considering the association of the LDH/WBC ratio together with compatible clinical features with COVID-19 disease at admission, the cut-off value higher than 25.64 could be used as a predictive tool to classify infected or uninfected patients and help in the medical management of patients in a provincial hospital. Furthermore, the result of the LDH/WBC ratio can help to prioritize those patients who urgently require the rRT-PCR test to be carried out over those who can wait, thus improving the utilization of technical resources of the chemical clinical laboratory. However, information about early prediction factors for positive cases is relatively limited and further investigation is needed.

This present study has several limitations. Firstly, our study was a single-center retrospective study, so external validation is required with larger samples and multicenter studies. Secondly, laboratory data was obtained without consulting the patient’s medical history and without taking into account their comorbidities or any other concomitant chronic medical illness. The LDH/WBC ratio would be better adjusted taking into account the patient’s underlying pathology. In future studies, it would be interesting to establish different cut-off points depending on the clinical characteristics of the patient. Thirdly, some negative rRT-PCR test cases could be erroneously classified because the viral load depends on the type of sample collected and the severity of the symptoms [28], [29]. Finally, the sample size was relatively small, which may have some impact on the statistical results.

## Conclusions

Many studies have shown that routine laboratory parameters are capable of discriminating between severe and nonsevere cases or determine the risk of mortality in COVID-19-infected patients. However, rRT-PCR continues to be the gold standard and the most reliable method for the differentiation between negative and COVID-19 positive pneumonia, but all the steps take a long time and spend the already limited medical resources. Realistically, the novelty of this study is focusing on the management of technical resources in a pandemic period when the number of infected patients increases dramatically and the performance of rRT-PCR test is limited by the shortage of reagents and laboratory staff. In this critical scenario, LDH/WBC ratio in combination with compatible symptoms of COVID-19 disease could predict more effectively those patients affected by the disease with a high probability (80.5%). In this way, LDH/WBC ratio can help to prioritize rRT-PCR tests for infected patients and enhance the assistance at collapsed Emergency Department.
